# Supplementary material for: Mutations and Differential Transcription of Mating-Type and Pheromone Receptor Genes in Hirsutella sinensis and the Natural Cordyceps sinensis Insect-Fungi Complex
Source: Biology (Basel). 2024 Aug 18;13(8):632. doi: 10.3390/biology13080632 (PMC11351167; doi:10.3390/biology13080632)
Supplement: Supplementary file 1 [file biology-13-00632-s001.zip › biology-3072190-supplementary.pdf]

Mutations and differential transcription of mating-type and pheromone receptor genes in *Hirsutella sinensis* and the natural *Cordyceps sinensis* insect–fungi complex

Xiu-Zhang Li, Meng-Jun Xiao, Yu-Ling Li, Ling Gao, Jia-Shi Zhu*

State Key Laboratory of Plateau Ecology and Agriculture, Qinghai Academy of Animal and Veterinary Sciences, Qinghai University, Xining, Qinghai, China

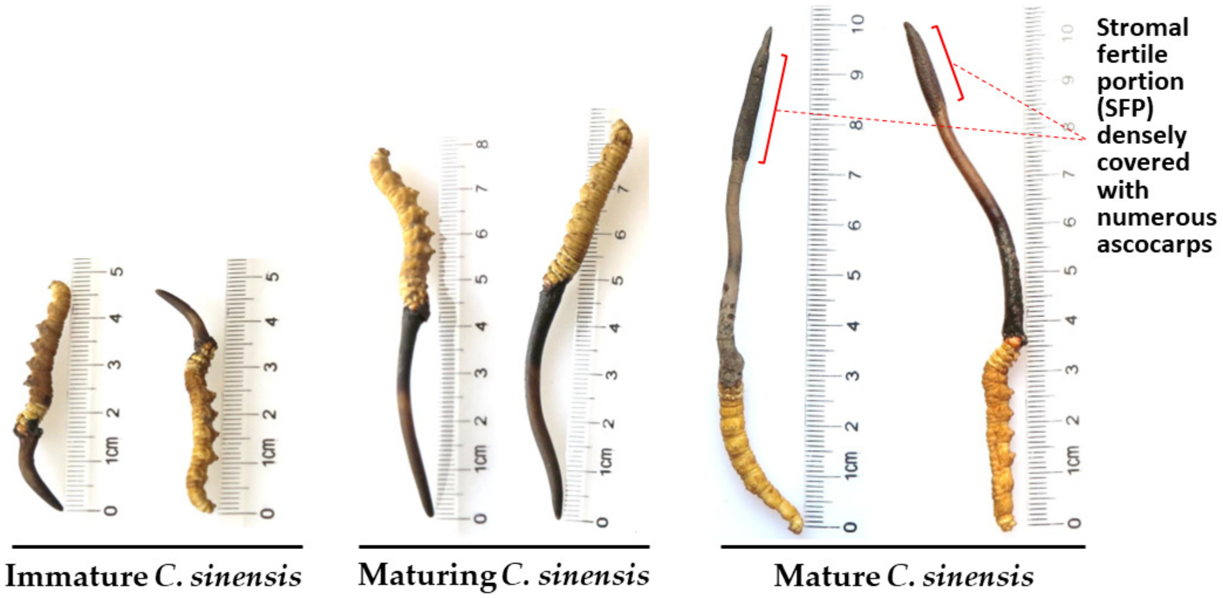

Figure S1. Images of immature, maturing, and mature natural *C. sinensis* insect-fungal complexes (modified from [50]). The right two pictures show mature *C. sinensis* specimens with the expanded stromal fertile portion (SFP) close to the tip of the *C. sinensis* stroma, which is densely covered with numerous ascocarps.

```
JQ325153      1        CCGTGCGACCATGGCCAATCCCATCAACATGATTCCCAATCCTCAGTGGAATGCGACTGACTACGAAGCGATCTGG 76
FJ654187      1    TCAG---------------------------------------------------------------------------- 80
JQ325237      1        ---------------------------------------------------------------------------- 76
KM197536      1        ---------G----------------------------------------------T------------------- 76
GCQL01020543  1157 TCAG---------------------------------------------------------------------------- 1078
OSIN7649      1                  ------------------------------------------------------------------ 66
(translation=)                    M  A  N  P  I  N  M  I  P  N  P  Q  W  N  A  T  D  Y  E  A  I  W
(variate aa=)                                                                I

JQ325153      77   AAAGGCCTCGAGGCACAGGTCAATCCTTTCTCGCAGATTCTCTGCCTGGAGGGGATTTTTCCGCCAGCTCGACGATGC 156
FJ654187      81   -------------------------------------------------------------------------------- 160
JQ325237      77   -----T--------------------------------C-------------------------------------GC-- 156
KM197536      77   ---------------------------------------------T---------------------------------- 156
GCQL01020543  1077 -------------------------------------------------------------------------------- 998
OSIN7649      67   -------------------------------------------------------------------------------- 146
(translation=)      K  G  L  E  A  Q  V  N  P  F  S  Q  I  L  C  L  E  G  D  F  F  R  Q  L  D  D  A
(variate aa=)                                                                                   G

JQ325153      157  TGCGAAGCTGTTCATTGCTCGGAAGCTCATGTAAGTTTGATGTTCACCGCAACTCGTCACACCTTCCTAATCGAATCTAC 236
FJ654187      161  -------------------------------TAA---TGA---------------------------TAA---------- 240
JQ325237      157  C--------------------C---------TAA---TGA---------------------------TAAC--------- 236
KM197536      157  ---A-----------------C---------TAA---TGA---------------------------TAAC--------- 236
GCQL01020543  997  -------------------------------                                                  968
OSIN7649      147  -------------------------------                                                  176
(translation=)       A  K  L  F  I  A  R  K  L  M

JQ325153      237  AGGGAACACGTTCAGGAGTCAGTCCTGTATGTCAATGACGGCAATGGACCCGATCGCGTCTACCTTGGAGCTCCCCGACA 316
FJ654187      241  -------------------------------------------------------------------------------- 320
JQ325237      237  -------------------------------------------------------------------------------- 316
KM197536      237  -------------------------------------------------------------------------------- 316
GCQL01020543  967    ------------------------------------------------------------------------------ 890
OSIN7649      177    ------------------------------------------------------------------------------ 254
(translation=)         E  H  V  Q  E  S  V  L  Y  V  N  D  G  N  G  P  D  R  V  Y  L  G  A  P  R  H

JQ325153      317  TTTTGTCGTTGGTGGTGGCATGATTCTCCAGATTTCTGGCTACGCGCCGTACTGGATCCGACGTTCCGTGTCGAAAGTCG 396
FJ654187      321  -------------------------------------------------------------------------------- 400
JQ325237      317  --------------------------------------------------------------------C----------- 396
KM197536      317  -------------------------------------------------------------------------------- 396
GCQL01020543  889  -------------------------------------------------------------------------------- 810
OSIN7649      253  -------------------------------------------------------------------------------- 334
(translation=)       F  V  V  G  G  G  M  I  L  Q  I  S  G  Y  A  P  Y  W  I  R  R  S  V  S  K  V
(variate aa=)                                                                              A

JQ325153      397  TTACTGCAACAGTGCTCGCGCCTCCCTCGCCCAAGGATATCAAGATCCCTCGTCCTCCCAACGCGTACATCTTGTACCGT 476
FJ654187      401  -------------------------------------------------------------------------------- 480
JQ325237      397  ----------G-----T--------------------------------------------------------------- 476
KM197536      397  ----C-----G--------------------------------------------------------------------- 476
GCQL01020543  809  -------------------------------------------------------------------------------- 730
OSIN7649      335  -------------------------------------------------------------------------------- 414
(translation=)     V  T  A  T  V  L  A  P  P  S  P  K  D  I  K  I  P  R  P  P  N  A  Y  I  L  Y  R

JQ325153      477  AAGGAGCGCCACCATCATGTCAAGGATGCAAATCCTGGCATCACGAACAACGAGATTTGTAAGTTTCATAGCCGCTCCTC 556
FJ654187      481  ---------------T-------------------------T---------------------------------------- 560
JQ325237      477  -------------------------------------------------------------------------------- 556
KM197536      477  -------------------------------------------------------------------------------- 556
GCQL01020543  739  ----------------------------------------------------------                       672
OSIN7649      315  ----------------------------------------------------------                       472
(translation=)      K  E  R  H  H  H  V  K  D  A  N  P  G  I  T  N  N  E  I
(variate aa=)                       Y

JQ325153      557  CTTTCTGCCATCGTGTTCTAATGTCTCTCTCAGCCCAAATCTTGGGCAAAGCTTGGAACATGGAGTCGAACGACGTCAGA 636
FJ654187      561  -------------------------------------------------------------------------------- 640
JQ325237      557  -------------------------------------------------------------------------------- 636
KM197536      557  -------------------------------------------A-----------------A------------------ 636
GCQL01020543  671                                   ----------------------------------------------- 625
OSIN7649      473                                   ----------------------------------------------- 519
(translation=)                                    S  Q  I  L  G  k  A  W  N  M  E  S  N  D  V  R
(variate aa=)                                                                I

JQ325153      637  CAGAAGTACAAGGACATGTCTCAGCAAGTCAAGCAAGCTCTCCTGGAGAAGCACCCAGACTACCAGTATAAACCGCGTCG 716
FJ654187      641  --------------------------------------------------------------------C----------- 720
JQ325237      637  --------------------------------------------------------------------C----------- 716
KM197536      637  -------------------------G------------------------------------------C----------- 716
GCQL01020543  624  --------------------------------------------------------------------C----------- 545
OSIN7649      520  --------------------------------------------------------------------C----------- 599
(translation=)      Q  K  Y  K  D  M  S  Q  Q  V  K  Q  A  L  L  E  K  H  P  D  Y  Q  Y  K  P  R  R
(variate aa=)                               R

JQ325153      717  TCCTTGCGAGCGCCGGCGCCGTCGTCGTGCCAGTCCGAACCAAAACCCGAAGCAATCTACGTCGAGAAATGCCGCTACTA 796
FJ654187      721  -------------------------------------------------------------------------------- 800
JQ325237      717  ---A-----------A------------A--------------------------------------------------- 796
KM197536      717  ---------------------------------C---------------------------------------------- 796
GCQL01020543  544  -------------------------------------------------------------------------------- 465
OSIN7649      600  -------------------------------------------------------------------------------- 679
(translation=)       P  C  E  R  R  R  R  R  R  A  S  P  N  Q  N  P  K  Q  S  T  S  R  N  A  A  T

JQ325153      797  GGGACGCCGCGATCTCGAGTGAAGATACTTCTACTGCCACCGGAGATACCAACACTGCGAATGGTTTCTGAGCGTCG    873
FJ654187      801  --------------------------------------------------------------------TAA------    877
JQ325237      797  --A---------------CC--G-----------------A--------A------------------TGA------    873
KM197536      781  ------------------CC----------------GG------------------------------TAA------    873
GCQL01020543  464  --------------------------------------------------------------------TGA------AAC 391
OSIN7649      664  --------------------------------------------------------------------TGA          750
(translation=)     R  D  A  A  I  S  S  E  D  T  S  T  A  T  G  D  T  N  T  A  N  G  F
(variate aa=)         N              T                 G

GCQL01020543  390  TTCTGCACTCTAGCACTGAGAATCACGTTCCCTCACGACAGCGACTGAAACTGAAAGTGAAAGACGGTCCAAGGCACAAG 311

GCQL01020543  310  CATACCGACAAGAAAATAAAAAAAAACTAAGCAATCCTTCTTTTACAACACACGGTTCTAGATGCGAATACATACCCCGA 231

GCQL01020543  230  GCTCCAAAATTGAGACTCGAAATAGTGCATCGACCCCACTTCCGTCACGATTCACGAATCCGTTTACGAACACCATCAAA 151

GCQL01020543  150  TATACCCCGCTGAAAACACCAGTTTATCAAAGCAGTTACGAAGCTGCAAGAAGAAGAACACCAGATGGGCTTCCCCCGGC 71

GCQL01020543  70   CCGAGCTGGCGCTTCAGGGGAGCGGCCAGGGTCACGCAGATAGTACATAGTTGCCGAAAAGGAG
```

Figure S2. Alignment of the MAT1-2-1 gene sequences with the transcriptome sequences obtained from *H. sinensis* Strain L0106 and natural *C. sinensis*. JQ325153, FJ654187, JQ325237 and KM197536 are the MAT1-2-1 gene sequences found in *H. sinensis* Strains GS09_121, CS2, XZ12_16, and XZ12_16, respectively [10,59]. GCQL01020543 is the transcriptome sequence obtained from the *H. sinensis* strain L0106 [52]. OSIN7649 is the metatranscriptome sequence obtained from natural *C. sinensis* collected from Deqin County, Yunnan Province, China [54]. The triplets shown in alternating brown and green indicate the open reading frame. The sequences in pink represent introns, and the underlined triplets "TAA" and "TGA" shown in blue are stop codons. The protein sequence was translated from the transcript sequences. The nucleotide and amino acid residues in red represent point mutations and variants, respectively. Hyphens indicate identical bases, and spaces denote unmatched sequence gaps.

```
KC437356       6530 ATGACGACAAGAAATGAGGTTATGCAGCGCTTGTCTTCTGTCCGAGCTGACGTTCTTCTGAACTTCCTCACGGACGATGC 6609
GAGW01008880
OSIN7648       1    -------------------------------------------------------------------------------- 80
(translation=)       M  T  T  R  N  E  V  M  Q  R  L  S  S  V  R  A  D  V  L  L  N  F  L  T  D  D  A

KC437356       6610 AATTTTCCAGCTTGCGTCCCGATATCACGAATCGACGACAGAGGCCGACGTTCTTACACCCGTGAGCACCGCAGCAGCAT 6689
GAGW01008880
OSIN7648       81   -------------------------------------------------------------------------------- 160
(translation=)        I  F  Q  L  A  S  R  Y  H  E  S  T  T  E  A  D  V  L  T  P  V  S  T  A  A  A

KC437356       6690 CTCGCGCCACTAGGCAGACCAAAGAAGCATCTTGTGATCGAGCGAAGCGACCTCTCAATGCCTTCATGGCATTCCGAAGT 6769
GAGW01008880
OSIN7648       161  ------------------------------------------------------------------------------   238
(translation=)      S  R  A  T  R  Q  T  K  E  A  S  C  D  R  A  K  R  P  L  N  A  F  M  A  F  R

KC437356       6770 ATGTTCATCCTCTTTCACGCACGTTGAAGTGGCTGACACGGACATTCTAGGTTACTACTTGAAGCTGTTTCCCGACGTGC 6849
GAGW01008880
OSIN7648       239                                                    ------------------------------ 288
(translation=)                                                        S  Y  Y  L  K  L  F  P  D  V

KC437356       6850 AGCAGAAGACCGCTTCTGGGTTTCTCACCACCTTGTGGCACAAAGACCCGTTCAGAAACAAGTGGGCGCTGATTGCGAAG 6929
GAGW01008880   1129                   ------------------------------------------------------------ 1088
OSIN7648       289  -------------------------------------------------------------------------------- 348
(translation=)      Q  Q  K  T  A  S  G  F  L  T  T  L  W  H  K  D  P  F  R  N  K  W  A  L  I  A  K

KC437356       6930 GTGTACTCCTTCGTGCGAGATCAGATTGGCAAGGACAAGGTTTCTCTATCATATTTCATGAGCCTTGCTTGTCCTACCAT 7009
GAGW01008880   1087 -------------------------------------------------------------------------------- 988
OSIN7648       359  -------------------------------------------------------------------------------- 428
(translation=)       V  Y  S  F  V  R  D  Q  I  G  K  D  K  V  S  L  S  Y  F  M  S  L  A  C  P  T  M

KC437356       7010 GACCATCATCGAGCCCGCTGCGTACCTGAACGCGCTTGGGTGGTGTGTCCAAGATGACGACGCTGGATCGCAGAAGCTAT 7089
GAGW01008880   987  -------------------------------------------------------------------------------- 908
OSIN7648       429  -------------------------------------------------------------------------------- 508
(translation=)        T  I  I  E  P  A  A  Y  L  N  A  L  G  W  C  V  Q  D  D  D  A  G  S  Q  K  L

KC437356       7090 TCCAAGACGAATCTTCTGCAAACCTGGACCAGTCCAGCTTGCTCTCGGCGGAATACCCCAGCACCGAAATCGAACTCTTG 7169
GAGW01008880   907  -------------------------------------------------------------------------------- 828
OSIN7648       509  -------------------------------------------------------------------------------- 588
(translation=)      F  Q  D  E  S  S  A  N  L  D  Q  S  S  L  L  S  A  E  Y  P  S  T  E  I  E  L  L

KC437356       7170 TCCGCCCTCGTCAACATTGGGTACTTTCCCGATCACGGCGCCGACCTTGTGGAGAGAATGGGATCCAGCCACAGTGGCAT 7249
GAGW01008880   827  -------------------------------------------------------------------------------- 748
OSIN7648       589  -------------------------------------------------------------------------------- 668
(translation=)       S  A  L  V  N  I  G  Y  F  P  D  H  G  A  D  L  V  E  R  M  G  S  S  H  S  G  I

KC437356       7250 CATGGCTCCGCGTGCCGCCAATTGCACTCCTCCAGTGTCCTACACGAAGGAAAAGATCGATTTCATCAACACAATCAGAA 7329
GAGW01008880   747  -------------------------------------------------------------------------------- 668
OSIN7648       669  -------------------------------------------------------------------------------- 748
(translation=)        M  A  P  R  A  A  N  C  T  P  P  V  S  Y  T  K  E  K  I  D  F  I  N  T  I  R

KC437356       7330 GCGATCCAGTTCAGGCGACAAAGGAGATCCTCGGTGATTGCTACGATGAAACCACAATCAAGCTTCTGGGTGTCAAGTCA 7409
GAGW01008880   667  -------------------------------------------------------------------------------- 588
OSIN7648       729  -------------------------------------------------------------------------------- 828
(translation=)      S  D  P  V  Q  A  T  K  E  I  L  G  D  C  Y  D  E  T  T  I  K  L  L  G  V  K  S

KC437356       7410 CACAATGTGGAGAGTGTTGACTCCATCACGCACTTGTCCATGCAACGCGAATATCAGGCTCCGCGATTTTTTTATGACTA 7489
GAGW01008880   587  -------------------------------------------------------------------------------- 508
OSIN7648       789  -----------------------------------                                              863
(translation=)       H  N  V  E  S  V  D  S  I  T  H  L  S  M  Q  R  E  Y  Q  A  P  R  F  F  Y  D  Y
(variant aa=)                                        F

KC437356       7490 CTCCGTCAGTTACGCAGGGATGGACTTCGGCGGTTCGAATGAGCCCGTGATGAACTTGAACAATCTCCCCGAGAACGAAA 7569
GAGW01008880   507  -------------------------------------------------------------------------------- 428
OSIN7648       864           ----------------------------------------------------------------------- 934
(translation=)        S  V  S  Y  A  G  M  D  F  G  G  S  N  E  P  V  M  N  L  N  N  L  P  E  N  E

KC437356       7570 CTTTCGACATCGACAGTCCTTTTGATCTCGATAAGATCCTTGGTCAATCGCAGTCAGAGGGCGAAAGAAGTGAGTGAAGC 7649
GAGW01008880   427  ---------------------------------------------------------------------            359
OSIN7648       935  ---------------------------------------------------------------------            1003
(translation=)      T  F  D  I  D  S  P  F  D  L  D  K  I  L  G  Q  S  Q  S  E  G  E  R

KC437356       7650 ATCCCATTCACAGATGTTTTTAGCTAACGGCGCCTAGCTTCTCATCTTCCTCCGAGTCCTCCACACAACCCTCTGGACGA 7729
GAGW01008880   358                                     ------------------------------------------- 316
OSIN7648       1004                                    ------------------------------------------- 1046
(translation=)                                         T  S  H  L  P  P  S  P  P  H  N  P  L  D  D

KC437356       7730 CTTTTACTTTGCGTTCTAGAGGGAAACGGGAGGCTCGACAGGAGCGGTATCAATTCGGGAGCTACAAAAAGGGGAGGCAC 7809
GAGW01008880   315  ----------------TAG------------------------------------------------------------- 236
OSIN7648       1047 ----------------TAG                                                              1065
(translation=)        F  Y  F  A  F

KC437356       7790 GATTCAAGACACGATTCAAAGCACATTTCAAGGCACGGTTCAAAGCACAATTCAAGAAATAGTTCAAGTTACAAGGCACG 7849
GAGW01008880   235  -------------------------------------------------------------------------------- 156

KC437356       7850 GTAGGGGACGAGGGACGATGCACGATTTCTCGTCCAGGAAGTGGAATTTTGCAGTGAATCCATCTTGCTCAGCGGGAGAT 7969
GAGW01008880   155  -------------------------------------------------------------------------------- 76

KC437356       7970 GGATCAAAAGACAGAGAAAGGGGGGCGTGCCGGGTAGACATGACAGGACCTAGGGCACAATCACTACTTCCGGTG      8044
GAGW01008880   75   ----------------C------A---------------------------------------------------      1
```

Figure S3. Alignment of the MAT1-1-1 genome sequences FC437356 and KR004003 of *H. sinensis* Strains CS68-2-1229 and GS09_225 and the transcriptome sequences of natural *C. sinensis*. FC437356 is the MAT1-1-1 gene of the *H. sinensis* strain CS68-2-1229 [37]. The sequence segments in pink represent introns I and II. The metatranscriptome assemblies OSIN7648 and GAGW01008880 are the MAT1-1-1 transcripts of natural *C. sinensis* specimens collected from Deqin County of Yunnan Province and Kangding County of Sichuan Province, China, respectively [53–54]. The triplets shown in alternating brown and green indicate the open reading frame. The underlined "TAG" triplets in blue represent stop codons. The protein sequence was translated from the transcript sequences. Amino acid residues in red represent variants. Hyphens indicate identical bases, and spaces denote unmatched sequence gaps.

The original plots for Panel 2A (ProtScale α-helix) of Figure 2

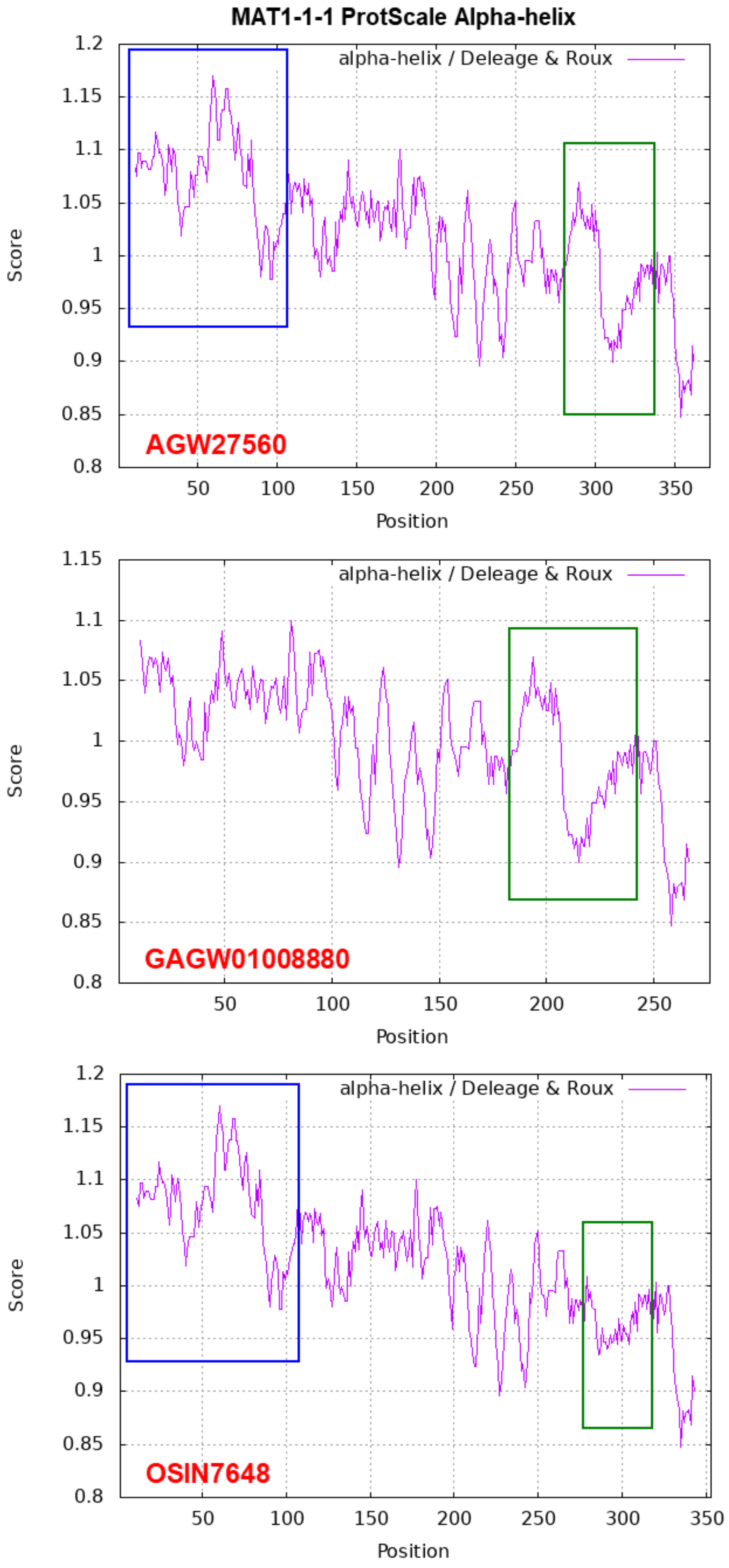

The original plots for Panel 2B (ProtScale β-sheet) of Figure 2

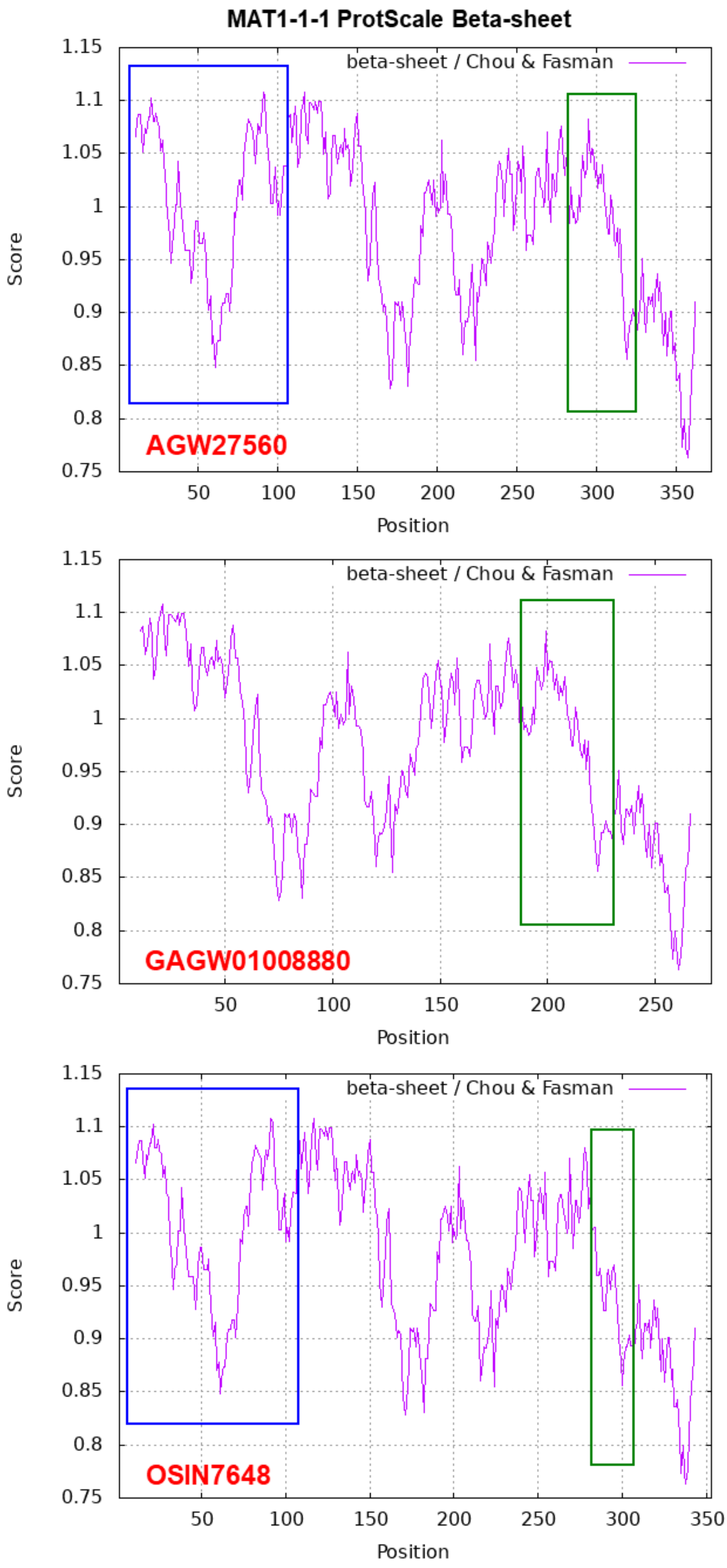

The original plots for Panel 2C (ProtScale β-turn) of Figure 2

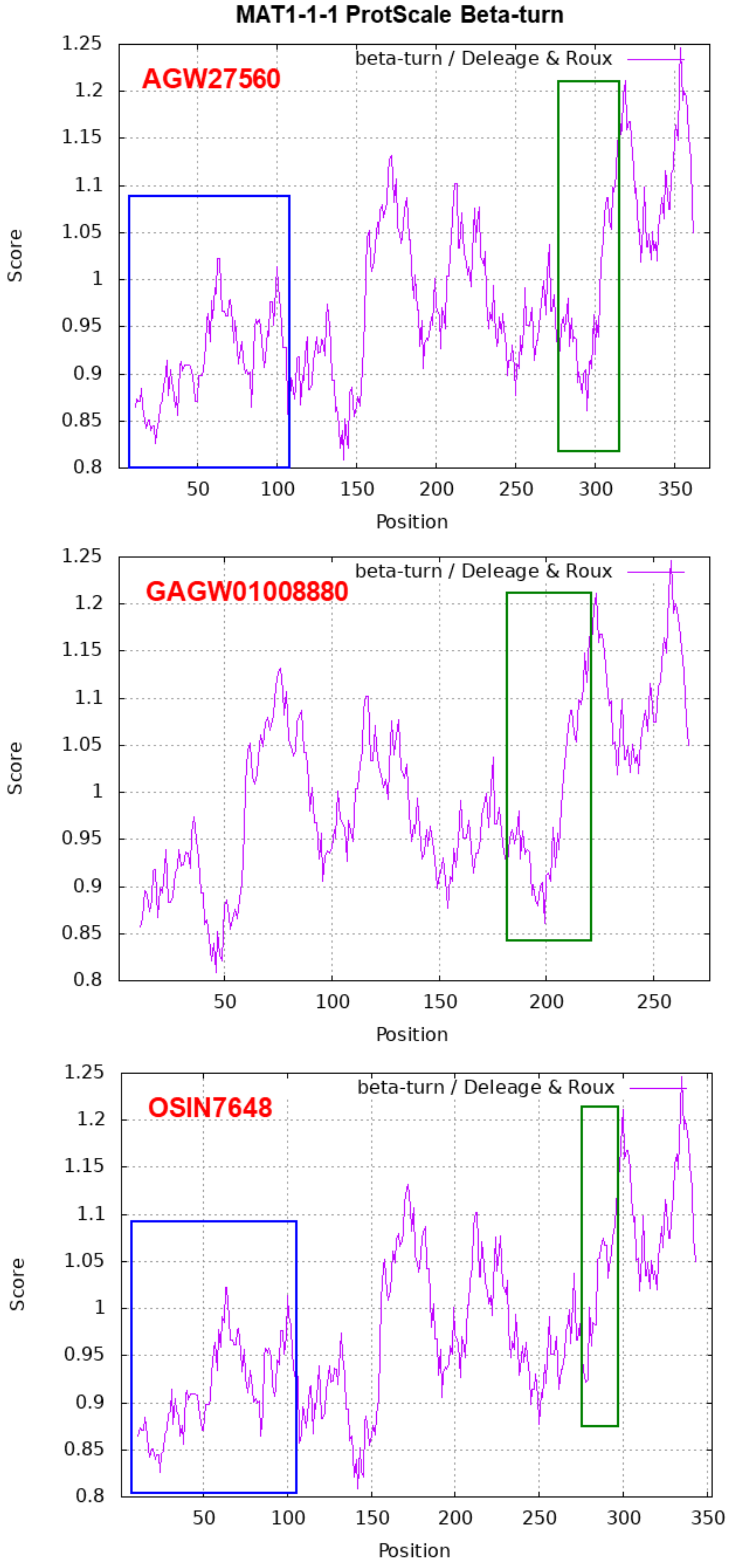

The original plots for Panel 2D (ProtScale coil) of Figure 2

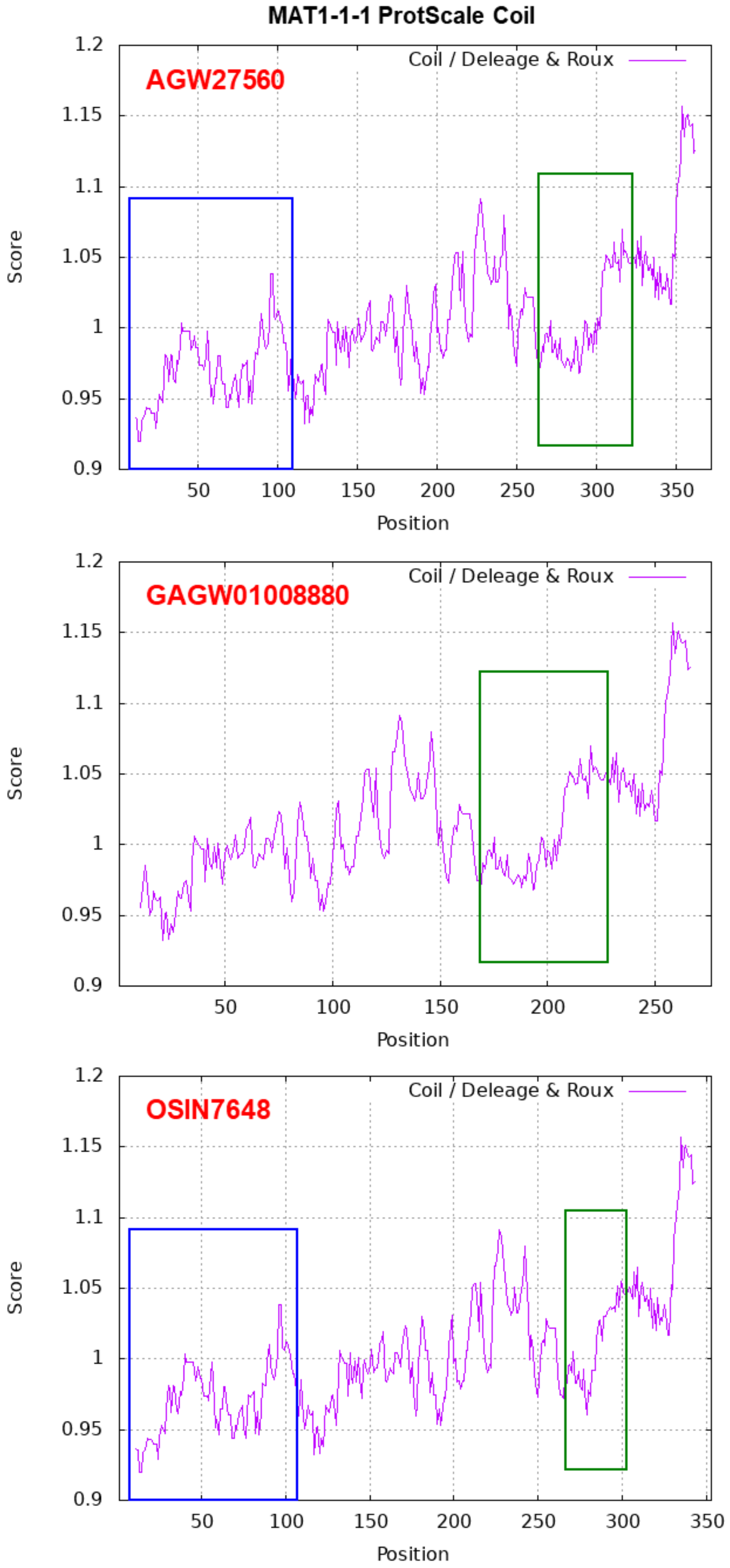

Figure S4. Original ExPASy ProtScale plots for the MAT1-1-1 protein α-helix (Figure 2 Panel 2A in the main text containing 3 plots: upper, middle, and lower plots), β-sheet (Figure 2 Panel 2B in the main text containing 3 plots), β-turn (Figure 2 Panel 2C in the main text containing 3 plots) and coil (Figure 2 Panel 2D in the main text containing 3 plots). The MAT1-1-1 protein sequence AGW27560 (372 aa; upper plots in all panels) of the *H. sinensis* strain CS68-2-1229 was compared with the metatranscriptome MAT1-1-1 sequences GAGW01008880 (276 aa; middle plots in all panels) and OSIN7648 (353 aa; lower plots in all panels) of natural *C. sinensis* (the original plots are included in Figure S4 in the supplementary file) [37,53–54]. The open boxes in green indicate the variable segment regions of the MAT1-1-1 proteins.

The original plots for Panel 8A (ProtScale Alpha-helix) of Figure 8

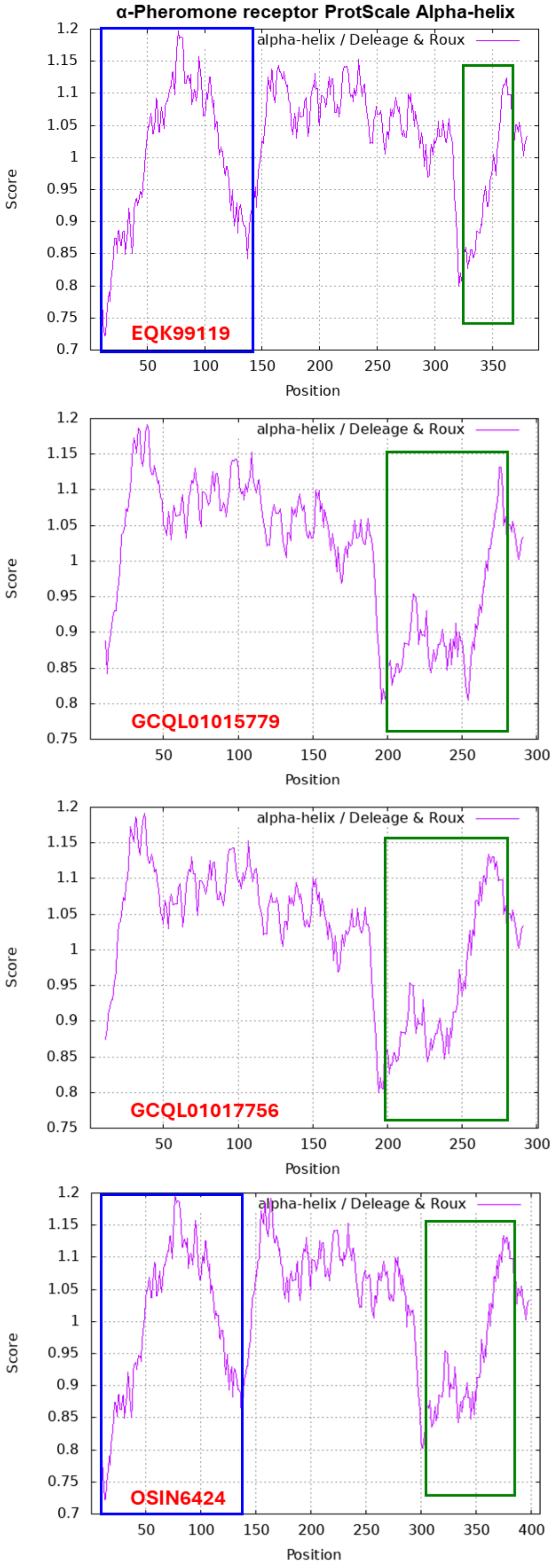

The original plots for Panel 8B (ProtScale Beta-sheet) of Figure 8

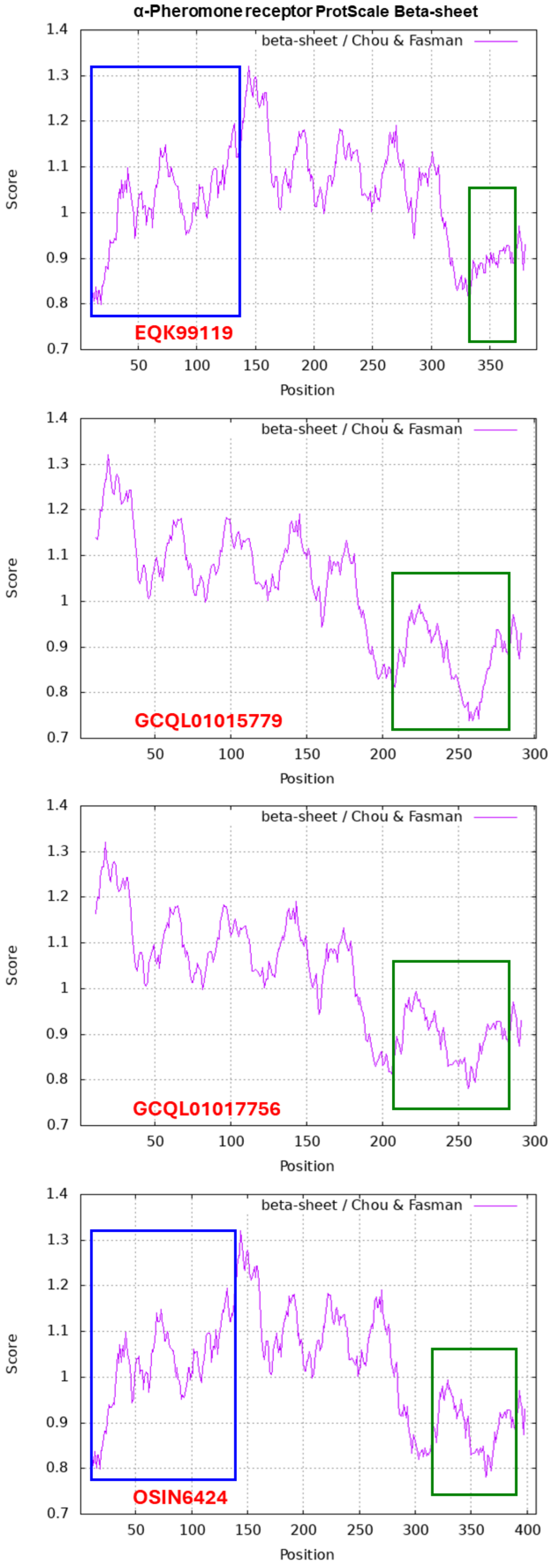

The original plots for Panel 8C (ProtScale Beta-turn) of Figure 8

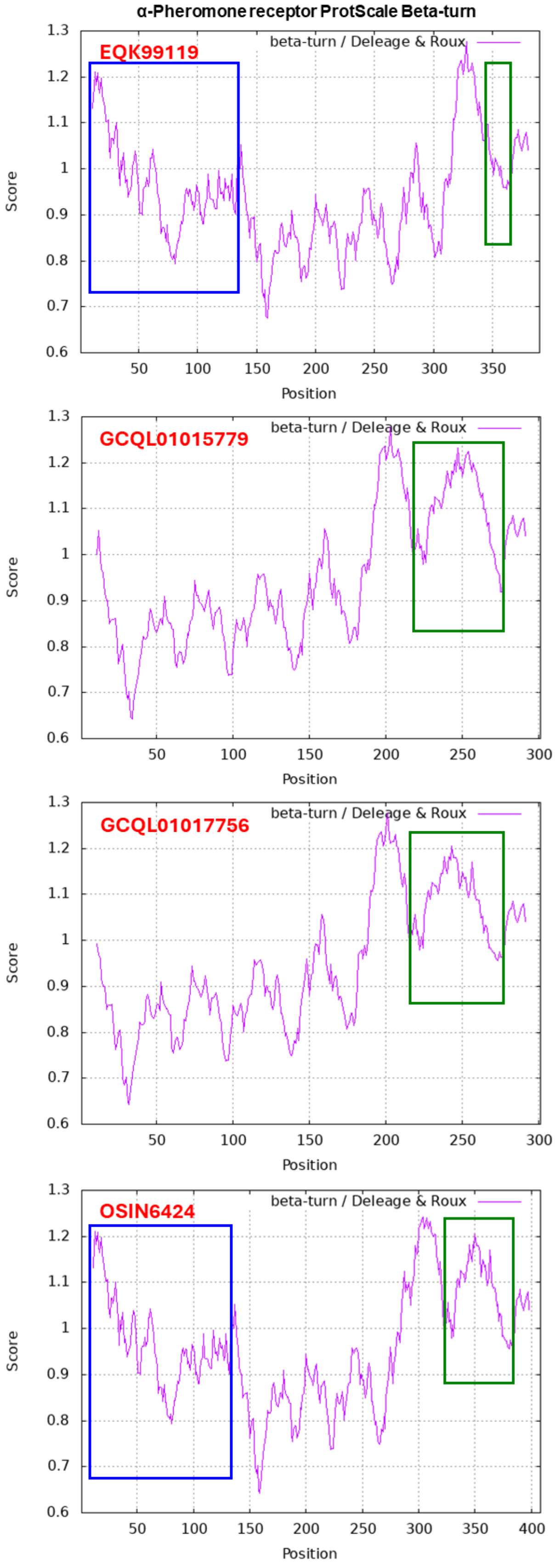

The original plots for Panel 8D (ProtScale Coil) of Figure 8

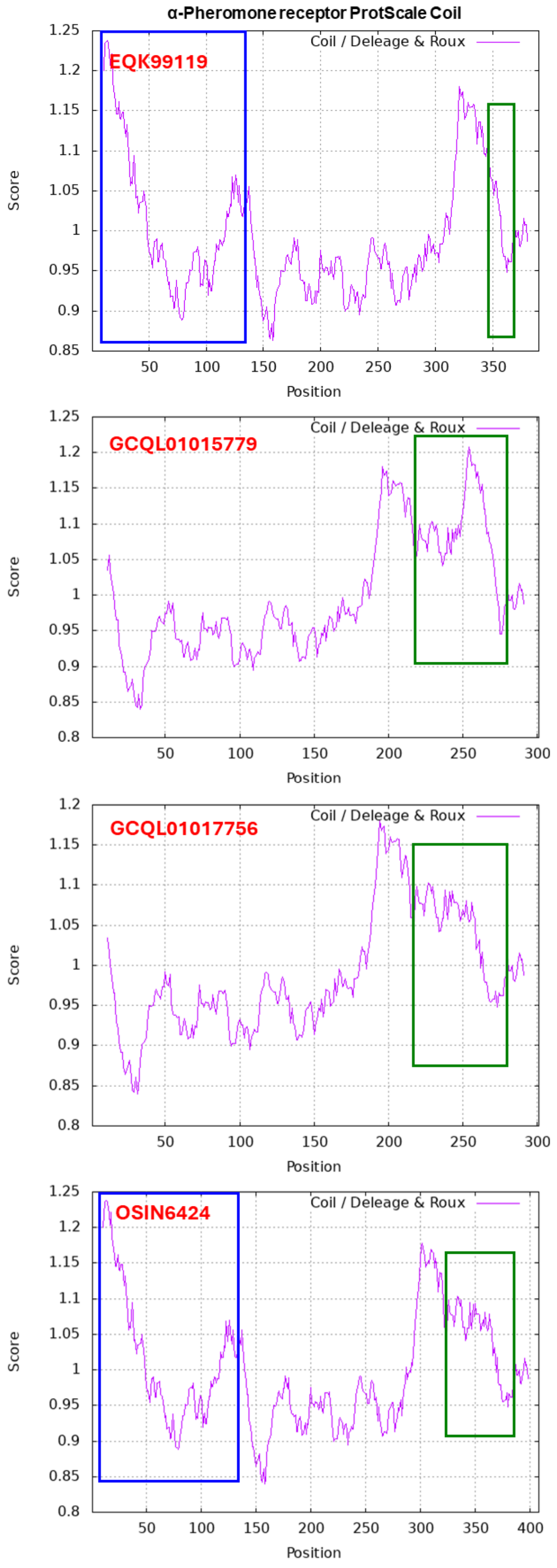

Figure S5. Original ExPASy ProtScale plots for the α-pheromone receptor protein α-helix (Figure 8 Panel 8A in the main text containing 4 plots: upper, middle, and lower plots), β-sheet (Figure 8 Panel 8B in the main text containing 4 plots), β-turn (Figure 8 Panel 8C in the main text containing 4 plots) and coil (Figure 8 Panel 8D in the main text containing 4 plots) of α-pheromone receptor proteins. The α-pheromone receptor protein sequence EQK99119 (390 aa; upper plots in all panels) of the *H. sinensis* strain Co18 was compared with the transcriptome and metatranscriptome α-pheromone receptor sequences and GCQL01017756 (301 aa; upper middle plots in all panels) and GCQL01015779 (301 aa; lower middle plots in all panels) of the *H. sinensis* strain L0106 and OSIN6424 (408 aa; lower plots in all panels) of natural *C. sinensis* [36,52,54]. The open boxes in green indicate the variable segment regions of the α-pheromone receptor proteins.

Table S1. Differential occurrence of the MAT1-1-1 and MAT1-2-1 genes in 237 *H. sinensis* strains.

**22 strains containing only the MAT1-1-1 gene**	**66 strains containing only the MAT1-2-1 gene**		**149 strains containing both the MAT1-1-1 and MAT1-2-1 genes**			
CS09-143	CC1406-203	XZ-CD-41	1229	NP10_1	SC09_47	XZ09_32
CS09-229	CS2	XZ-CD-59	Co18	NP10_2	SC09_57	XZ09_46
GS03	GS09_337	XZ-CD-64	CS6-251	QH01	SC09_65	XZ09_48
IOZ07	QH02	XZ-ML-191	CS18-266	QH03	SC09_77	XZ09_59
QH07_188	QH05	XZ-LZ05-6	CS25-273	QH04	SC09_87	XZ09_71
QH07_197	QH09_11	XZ-LZ06_1	CS26-277	QH06	SC09_97	XZ09_80
XZ05_3	QH09_187	XZ-LZ06_7	CS34-291	QH07	SC09_107	XZ09_95
XZ05_6	QH-LJ-214	XZ-LZ06_21	CS36-1294	QH08	SC09_117	XZ09_100
XZ07_H2	QH-LJ-236	XZ-LZ06_61	CS37-295	QH09_20L	SC09_128	XZ09_106
XZ07_108	QH-QL-206	XZ-LZ06_108	CS68-2-1228	QH09_33L	SC09_147	XZ09_113
XZ07_154	QH-QL-207	XZ-LZ07_30	CS68-2-1229	QH09_37	SC09_157	XZ09_118
XZ07_166	QH-YS-188	XZ-LZ07_64	CS68-5-1216	QH09_46	SC09_167	XZ10_7
XZ07_176	QH-YS-189	XZ-LZ07_H1	CS70-1208	QH09_56	SC09_180	XZ10_15
XZ07_180	QH-YS-196	XZ-LZ07_H2	CS70-1211	QH09_66	SC09_190	XZ10_17
XZ08_4	QH-YS-197A	XZ-NQ_74	CS70-1212	QH09_78	SC09_200	XZ10_23
XZ08_10	QH-YS-197B	XZ-NQ_80	CS71-1218	QH09_93	SC10_4	XZ12_1
XZ08_26	QH-YS-199	XZ-NQ_84	CS71-1219	QH09_122	SC10_18	XZ12_16
XZ08_59	SC-2	XZ-NQ_86	CS71-1220	QH09_131	SC10_21	XZ12_33
XZ08_A1	SC-3	XZ-NQ_92	CS76-1284	QH09_151	TB01	XZ12_43
XZ08_B1	SC-4	XZ-NQ_139	CS91-1291	QH09_164	TB02	YN01
YN07_6	SC-5	XZ-NQ_154	CS560-961	QH09_173	TB03	YN02
YN07_8	SC-7	XZ-NQ_155	CS561-964	QH09_201	TB04	YN03
	SCK05-4-3	XZ-NQ_156	GS01	QH09_210	TB05	YN09_3
	XZ07_11	XZ-NQ_166	GS02	QH10_1	TB06	YN09_6
	XZ07_46	XZ-NQ_176	GS04	QH10_4	TB07	YN09_22
	XZ08_33	XZ-NQ_180	GS05	QH10_7	TB08	YN09_51
	XZ08_38	XZ-SN_44	GS09_111	SC01	XZ05_2	YN09_61
	XZ-CD-A1	YN-1	GS09_121	SC02	XZ05_7	YN09_64
	XZ-CD-B1	YN-4	GS09_131	SC03	XZ05_8	YN09_72
	XZ-CD-4	YN-5	GS09_143	SC04	XZ05_12	YN09_81
	XZ-CD-10	YN-6	GS09_201	SC05	XZ06_124	YN09_85
	XZ-CD-26	YN-8	GS09_225	SC06	XZ06_152	YN09_89
	XZ-CD-30	ZJB12195	GS09_229	SC07	XZ06_260	YN09_96
			GS09_281	SC08	XZ07_133	YN09_101
			GS09_311	SC09_1	XZ08_24	YN09_140
			GS10_1	SC09_21	XZ08_56	
			GS10_10	SC09_36	XZ09_4	
			ID10_1	SC09_37	XZ09_15	

Table S2. Amino acid scales for ProtScale analysis (https://web.expasy.org/protscale/) to predict secondary structures (α-helices, β-turns, and coils [57] and β-sheets [56]) of proteins.

			Chemical-physical property	α-Helix	β-Sheet	β-Turn	Coil
Aspartic acid	Asp	D	Acidic	0.924	0.540	1.197	1.197
Glutamic acid	Glu	E	Acidic	1.504	0.370	1.149	0.761
Alanine	Ala	A	Aliphatic	1.489	0.830	0.788	0.824
Isoleucine	Ile	I	Aliphatic	1.003	1.600	0.240	0.886
Leucine	Leu	L	Aliphatic	1.236	1.300	0.670	0.810
Valine	Val	V	Aliphatic	0.990	1.700	0.387	0.772
Phenylalanine	Phe	F	Aromatic	1.195	1.380	0.624	0.797
Tryptophan	Trp	W	Aromatic	1.090	1.370	0.546	0.941
Tyrosine	Tyr	Y	Aromatic	0.787	1.470	0.795	1.109
Arginine	Arg	R	Basic	1.224	0.930	0.912	0.893
Histidine	His	H	Basic	1.003	0.870	0.970	1.068
Lysine	Lys	K	Basic	1.172	0.740	1.302	0.897
Asparagine	Asn	N	with polar neutral side chains	0.772	0.890	1.572	1.167
Cysteine	Cys	C	with polar neutral side chains	0.966	1.190	0.965	0.953
Glutamine	Gln	Q	with polar neutral side chains	1.164	1.100	0.997	0.947
Methionine	Met	M	with polar neutral side chains	1.363	1.050	0.436	0.810
Serine	Ser	S	with polar neutral side chains	0.739	0.750	1.316	1.130
Threonine	Thr	T	with polar neutral side chains	0.785	1.190	0.739	1.148
Glycine	Gly	G	Unique amino acids	0.510	0.750	1.860	1.251
Proline	Pro	P	Unique amino acids	0.492	0.550	1.415	1.540

Note: An amino acid scale was defined at https://web.expasy.org/protscale/ as follows:
An **amino acid scale** is defined by a numerical value assigned to each type of amino acid. The most frequently used scales are the hydrophobicity or hydrophilicity scales and the secondary structure conformational parameter scales, but many other scales exist, which are based on the different chemical and physical properties of the amino acids. This program provides 57 predefined scales entered from the literature.

Table S3. Percent similarity between the sequences of the ITS1, 5.8S, and/or ITS2 segments of the parental fungi (Genotype #1 *H. sinensis* AB067721 and the AB067719-type Group E fungus) and the segment sequences of the offspring variant Genotypes #13 (KT339190, in the semiejected ascospores) and #14 (KT339178, in the fully ejected ascospores) of *O. sinensis* (adapted from [5–6,15]).

	Percent similarity			
	ITS1-5.8S-ITS2	ITS1	5.8S	ITS2
	***vs.* Genotype #13 of *O. sinensis* (KT339190)**			
Genotype #1 AB067721 (*H. sinensis*)	86.3%	**100%**	94.8%	64.2%
Group E AB067719 fungus	88.2%	71.5%	**100%**	**99%**
	***vs.* Genotype #14 of *O. sinensis* (KT339178)**			
Genotype #1 AB067721 (*H. sinensis*)	87.7%	67.9%	94.9%	**100%**
Group E AB067719 fungus	89.2%	**100%**	**100%**	71.5%

[REFERENCES]

1. Zhu, J.-S., Halpern, G.M., Jones, K. The scientific rediscovery of an ancient Chinese herbal medicine: *Cordyceps sinensis*: Part I. J. Altern. Complem. Med. 1998a;4(3): 289–303. DOI: 10.1089/acm.1998.4.3-289
2. Zhu, J.-S., Halpern, G.M., Jones, K. The scientific rediscovery of a precious ancient Chinese herbal regimen: *Cordyceps sinensis*: Part II. J. Altern. Complem. Med. 1998b;4(4): 429–457. DOI: 10.1089/acm.1998.4.429

3. Zhu, J.-S., Li, C.-L., Tan, N.-Z., Berger, J.L., Prolla, T.A. Combined use of whole-gene expression profiling technology and mouse lifespan test in anti-aging herbal product study. Proc. 2011 New TCM Products Innovation and Industrial Development Summit, Hangzhou, China (Nov 27, 2011). pp. 443–448. https://xueshu.baidu.com/usercenter/paper/show?paperid=08341c17fa58cXZ8f85584b92572b90f75&site=xueshu_se
4. Ren, Y., Wan, D.-G., Lu, X.-M., Guo, J.-L. The study of scientific name discussion for TCM Cordyceps. LisShenzhen Med. Material Medica Res. 2013;24(9): 2211–2212.
5. Li, Y.-L., Li, X.-Z., Yao, Y.-S., Xie, W.-D., Zhu, J.-S. Molecular identification of *Ophiocordyceps sinensis* genotypes and the indiscriminate use of the Latin name for the multiple genotypes and the natural insect-fungi complex. Am J BioMed Sci. 2022b;14(3): 115–135. doi: 10.5099/aj220300115
6. Zhu, J.-S., Li, Y.-L. A Precious Transitional Chinese Medicine, *Cordyceps sinensis*: Multiple heterogeneous *Ophiocordyceps sinensis* in the insect-fungi complex. Lambert Academic Publishing, Saarbrüchen. Germany, 2017.
7. Kinjo, N., Zang, M. Morphological and phylogenetic studies on *Cordyceps sinensis* distributed in southwestern China. Mycoscience. 2001;42: 567–574. DOI: 10.1007/BF02460956
8. Stensrud, Ø., Schumacher, T., Shalchian-Tabrizi, K., Svegardenib, I.B., Kauserud, H. Accelerated nrDNA evolution and profound AT bias in the medicinal fungus *Cordyceps sinensis*. Mycol. Res. 2007;111: 409–415. DOI: 10.1016/j.mycres.2007.01.015
9. Xiao, W., Yang, J.-P., Zhu, P., Cheng, K.-D., He, H.-X., Zhu, H.-X., Wang, Q. Non-support of species complex hypothesis of *Cordyceps sinensis* by targeted rDNA-ITS sequence analysis. Mycosystema. 2009;28(6): 724–730.
10. Zhang, Y.-J., Xu, L.-L., Zhang, S., Liu, X.-Z., An, Z.-Q., Wàngmŭ, , Guo, Y.-L. Genetic diversity of *Ophiocordyceps sinensis*, a medicinal fungus endemic to the Tibetan Plateau: implications for its evolution and conservation. BMC Evol. Biol. 2009;9: 290. doi:10.1186/1471-2148-9-290
11. Zhu, J.-S., Gao, L., Li, X.-H., Yao, Y.-S., Zhou, Y.-J., Zhao, J.-Q., Zhou, Y.-J. Maturational alterations of oppositely orientated rDNA and differential proliferations of CG:AT-biased genotypes of *Cordyceps sinensis* fungi and *Paecilomyces hepiali* in natural *C. sinensis*. Am. J. Biomed. Sci. 2010;2(3): 217–238. doi: 10.5099/aj100300217
12. Chen, C.-S., Hseu, R.-S., Huang, C.-T. Quality control of *Cordyceps sinensis* teleomorph, anamorph, and Its products. Chapter 12, *in* (Shoyama, Y., Ed.) *Quality Control of Herbal Medicines and Related Areas*. InTech, Rijeka, Croatia, 2011; pp. 223–238. www.intechopen.com
13. Li, Y., Jiao, L., Yao, Y.-J. Non-concerted ITS evolution in fungi, as revealed from the important medicinal fungus *Ophiocordyceps sinensis*. Mol. Phylogenet. Evol. 2013;68: 373–379. doi: 10.1016/j.ympev.2013.04.010
14. Li, Y.-L., Gao, L., Yao, Y.-S., Wu, Z.-M., Lou, Z.-Q., Xie, W.-D., Wu, J.-Y., Zhu, J.-S. Altered GC- and AT-biased genotypes of *Ophiocordyceps sinensis* in the stromal fertile portions and ascospores of natural *Cordyceps sinensis*. PLoS ONE. 2023b;18(6): e0286865. https://doi.org/10.1371/journal.pone.0286865
15. Li, Y.-L., Li X-Z, Yao, Y.-S., Wu, Z.-M., Gao, L., Tan N-Z, Lou, Z.-Q., Xie, W.-D., Wu, J.-Y., Zhu, J.-S. Differential cooccurrence of multiple genotypes of *Ophiocordyceps sinensis* in the stromata, stromal fertile portion (ascocarps) and ascospores of natural *Cordyceps sinensis*. PLoS ONE. 2023c;18(3): e0270776. DOI: 10.1371/journal.pone.0270776
16. Mao, X.-M., Zhao, S.-M., Cao, L., Yan, X., Han, R.-C. The morphology observation of *Ophiocordyceps sinensis* from different origins. J. Environ. Entomol. 2013;35(3): 343–353.
17. Li, C.-L. A study of *Tolypocladium sinense* C.L. Li. sp. nov. and cyclosporin production. Acta Mycol. Sinica. 1988;7: 93–98.
18. Li, X., Wang, F., Liu, Q., Li, Q.-P., Qian, Z.-M., Zhang, X.-L., Li, K., Li, W.-J., Dong, C.-H. Developmental transcriptomics of Chinese cordyceps reveals gene regulatory network and expression profiles of sexual development-related genes. BMC Genomics. 2019;20: 337. https://doi.org/10.1186/s12864-019-5708-z
19. Dai, R.-Q., Lan, J.-L., Chen, W.-H., Li, X.-M., Chen, Q.-T., Shen, C.-Y. Discovery of a new fungus *Paecilomyces hepiali* Chen & Dai. Acta Agricult. Univ. Pekin. 1989;15(2): 221–224.
20. Jiang, Y., Yao, Y.-J. A review for the debating studies on the anamorph of *Cordyceps sinensis*. Mycosistema. 2003;22(1): 161–176.
21. Leung, P.-H., Zhang, Q.-X., Wu, J.-Y. Mycelium cultivation, chemical composition and antitumour activity of a *Tolypocladium* sp. fungus isolated from wild *Cordyceps sinensis*. J. Appl. Microbiol. 2006;101: 275–283. DOI: 10.1111/j.1365-2672.2006.02930.x
22. Zhu, J.-S., Guo, Y.-L., Yao, Y.-S., Zhou, Y.-J., Lu, J.-H., Qi, Y., Chen, W., Zheng, T.-Y., Zhang, L., Wu, Z.-M., Zhang, L.-J., Liu, X.-J., Yin, W.-T. Maturation of *Cordyceps sinensis* associates with co-existence of *Hirsutella sinensis* and *Paecilomyces hepiali* DNA and dynamic changes in fungal competitive proliferation predominance and chemical profiles. J. Fungal Res. 2007;5(4): 214–224.
23. Zhang, Y.-J., Sun, B.-D., Zhang, S., Wàngmŭ, Liu, X.-Z., Gong, W.-F. Mycobiotal investigation of natural *Ophiocordyceps sinensis* based on culture-dependent investigation. Mycosistema. 2010;29(4): 518–527.
24. Barseghyan, G.S., Holliday, J.C., Price, T.C., Madison, L.M., Wasser, S.P. Growth and cultural-morphological characteristics of vegetative mycelia of medicinal caterpillar fungus *Ophiocordyceps sinensis* G.H. Sung *et al*. (Ascomycetes) Isolates from Tibetan Plateau (P.R.China). Intl. J. Med. Mushrooms. 2011;13(6): 565–581. DOI: 10.1615/intjmedmushr.v13.i6.90
25. Xia, F., Liu, Y., Shen, G.-L., Guo, L.-X., Zhou, X.-W. Investigation and analysis of microbiological communities in natural *Ophiocordyceps sinensis*. Can. J. Microbiol. 2015;61: 104–111. DOI: 10.1139/cjm-2014-0610
26. Wang, J.-B., St. Leger, R.J., Wang, C.-S. Advances in Genomics of Entomopathogenic Fungi. Advan. Genetics. 2016;94: 1–39. DOI: 10.1016/bs.adgen.2016.01.002
27. Meng, Q., Yu, H.-Y., Zhang, H., Zhu, W., Wang, M.-L., Zhang, J.-H., Zhou, G.-L., Li, X., Qin, Q.-L., Hu, S.-N., Zou, Z. Transcriptomic insight into the immune defenses in the ghost moth, *Hepialus xiaojinensis*, during an *Ophiocordyceps sinensis* fungal infection. Insect Biochem. Mol. Biol. 2015;64: 1–15. DOI: 10.1016/j.ibmb.2015.06.014

28. Lu, H.-L., St Leger, R.J. Chapter Seven - Insect Immunity to Entomopathogenic Fungi, Editors: Lovett B, St. Leger RJ. Advanc Genet, Academic Press, 2016. Vol. 94; pp. 251-85.
29. Wang, Y., Stata, M., Wang, W., Stajich, J.E., White, M.M., Moncalvo, J.M. Comparative genomics reveals the core gene toolbox for the fungus-insect symbiosis. mBio. 2018;9: e00636-18. DOI: 10.1128/mBio.00636-18
30. Sung, G.-H., Hywel-Jones, N.L., Sung, J.-M., Luangsa-ard, J.J., Shrestha, B., Spatafora, J.W. Phylogenetic classification of *Cordyceps* and the clavicipitaceous fungi. Stud. Mycol. 2007;57: 5–59. DOI: 10.3114/sim.2007.57.01
31. Zhang, Y.-J., Li, E.-W., Wang, C.-S., Li, Y.-L., Liu, X.-Z. *Ophiocordyceps sinensis*, the flagship fungus of China: terminology, life strategy and ecology. Mycol. 2012;3(1): 2–10. https://www.tandfonline.com/doi/full/10.1080/21501203.2011.654354
32. Debuchy, R., Turgeo, B.G. Mating-Type Structure, Evolution, and Function in Euascomycetes. In (eds. Kües U. & Fischer, R.) Growth, Differentiation and Sexuality. Springer, 2006; pp. 293–323.
33. Jones, S.K., Bennett, R.J. Fungal mating pheromones: choreographing the dating game. Fungal Genet. Biol. 2011;48(7): 668–676. doi: 10.1016/j.fgb.2011.04.001
34. Zheng, P., Wang, C.-S. Sexuality Control and Sex Evolution in Fungi. Sci Sinica Vitae. 2013;43:1090–1097.
35. Wilson, A.M., Wilken, P.M., van der Nest, M.A., Steenkamp, E.T., Wingfield, M.J., Wingfield, B.D Homothallism: an umbrella term for describing diverse sexual behaviours. IMA Fungus. 2015;6(1): 207–214. DOI: 10.5598/imafungus.2015.06.01.13
36. Hu, X., Zhang, Y.-J., Xiao, G.-H., Zheng, P., Xia, Y.-L., Zhang, X.-Y., St Leger, R.J., Liu, X.-Z., Wang, C.-S. Genome survey uncovers the secrets of sex and lifestyle in caterpillar fungus. Chin. Sci. Bull. 2013;58: 2846–2854. doi: 10.1007/s11434-013-5929-5
37. Bushley, K.E., Li, Y., Wang, W.-J., Wang, X.-L., Jiao, L., Spatafora, J.W., Yao, Y.-J. Isolation of the MAT1-1 mating type idiomorph and evidence for selfing in the Chinese medicinal fungus *Ophiocordyceps sinensis*. Fungal Biol. 2013;117(9): 599–610. DOI: 10.1016/j.funbio.2013.06.001
38. Zhang, S., Zhang, Y.-J., Liu, X.-Z., Wen, H.-A., Wang, M., Liu, D.-S. Cloning and analysis of the MAT1-2-1 gene from the traditional Chinese medicinal fungus *Ophiocordyceps sinensis*. Fungal Biol. 2011;115: 708–714. DOI: 10.1016/j.funbio.2011.05.004
39. Zhang, S., Zhang, Y.-J. Molecular evolution of three protein-coding genes in the Chinese caterpillar fungus *Ophiocordyceps sinensis*. Microbiol. China. 2015;42(8): 1549–1560.
40. Wei, X.-L., Yin, X.-C., Guo, Y.-L., Shen, N.-Y., Wei, J.-C. Analyses of molecular systematics on *Cordyceps sinensis* and its related taxa. Mycosystema. 2006;25(2): 192–202.
41. Engh IB. Molecular phylogeny of the *Cordyceps-Tolypocladium* complex. Candidate scientific thesis, Department of Biology, University of Oslo, 1999.
42. Stensrud, Ø., Hywel-Jones, N.L., Schumacher, T. Towards a phylogenetic classification of *Cordyceps*: ITS nrDNA sequence data confirm divergent lineages and paraphyly. Mycol. Res. 2005;109: 41–56. DOI: 10.1017/s095375620400139x
43. Li, Y., Hsiang, T., Yang, R.-H., Hu, X.-D., Wang, K., Wang, W.-J., Wang, X.-L., Jiao, L., Yao, Y.-J. Comparison of different sequencing and assembly strategies for a repeat-rich fungal genome, *Ophiocordyceps sinensis*. J. Microbiol. Methods. 2016a;128: 1–6. DOI: 10.1016/j.mimet.2016.06.025
44. Jin, L.-Q., Xu, Z.-W., Zhang, B., Yi, M., Weng, C.-Y., Lin, S., Wu, H., Qin, X.-T., Xu, F., Teng, Y., Yuan, S.-J., Liu, Z.-Q., Zheng, Y.-G. Genome sequencing and analysis of fungus *Hirsutella sinensis* isolated from *Ophiocordyceps sinensis*. AMB Expr. 2020;10: 105. https://doi.org/10.1186/s13568-020-01039-x
45. Liu, J., Guo, L.-N., Li, Z.-W., Zhou, Z., Li, Z., Li, Q., Bo, X.-C., Wang, S.-Q., Wang, J.-L., Ma, S.-C., Zheng, J., Yang, Y. Genomic analyses reveal evolutionary and geologic context for the plateau fungus *Ophiocordyceps sinensis*. Clin. Med. 2020;15: 107–119. https://doi.org/10.1186/s13020-020-00365-3
46. Shu, R.-H., Zhang, J.-H., Meng, Q., Zhang, H., Zhou, G.-L., Li, M.-M., Wu, P.-P., Zhao, Y.-N., Chen, C., Qin, Q.-L. A new high-quality draft genome assembly of the Chinese cordyceps *Ophiocordyceps sinensis*. Genome Biol. Evol. 2020;12(7): 1074–1079. DOI: 10.1093/gbe/evaa112
47. Li, X.-Z., Li, Y.-L., Yao, Y.-S., Xie, W.-D., Zhu, J.-S. Further discussion with Li *et al*. (2013, 2019) regarding the "ITS pseudogene hypothesis" for *Ophiocordyceps sinensis*. Mol. Phylogenet. Evol. 2020;146: 106728. https://doi.org/10.1016/j.ympev.2019.106728
48. Li, X.-Z., Li, Y.-L., Zhu, J.-S. Differential transcription of mating-type genes during sexual reproduction of natural *Cordyceps sinensis*. Chin. J. Chin. Materia Medica. 2023a;48(10): 2829–2840. DOI: 10.19540/j.cnki.cjcmm.20230213.102
49. Zhang, S., Zhang, Y.-J., Shrestha, B., Xu, J.-P., Wang, C.-S., Liu, X.-Z. *Ophiocordyceps sinensis* and *Cordyceps militaris*: research advances, issues and perspectives. Mycosystema. 2013;32:577–597.
50. Yao, Y.-S., Gao, L., Li, Y.-L., Ma, S.-L., Wu, Z.-M., Tan, N.-Z., Wu, J.-Y., Ni, L.-Q., Zhu, J.-S.. Amplicon density-weighted algorithms for analyzing dissimilarity and dynamic alterations of RAPD polymorphisms of *Cordyceps sinensis*. Beijing Da Xue Xue Bao Yi Xue Bao. 2014;46: 618–628. (http://xuebao.bjmu.edu.cn/EN/Y2014/V46/I4/618)
51. Liang, Z.-Q., Han, Y.-F., Liang, J.-D., Dong, X., Du, W. Issues of concern in the studies of *Ophiocordyceps sinensis*. Microbiol. Chin. 2010;37(11): 1692–1697.
52. Liu, Z.-Q., Lin, S., Baker, P.J., Wu, L.-F., Wang, X.-R., Wu, H., Xu, F., Wang, H.-Y., Brathwaite, M.E., Zheng, Y.-G. Transcriptome sequencing and analysis of the entomopathogenic fungus *Hirsutella sinensis* isolated from *Ophiocordyceps sinensis*. BMC Genomics. 2015;16: 106–123. DOI: 10.1186/s12864-015-1269-y
53. Xiang, L., Li, Y., Zhu, Y., Luo, H., Li, C., Xu, X., Sun, C., Song, J.-Y., Shi, L.-H., He, L., Sun, W., Chen, S.-L. Transcriptome analysis of the *Ophiocordyceps sinensis* fruiting body reveals putative genes involved in fruiting body development and cordycepin biosynthesis. Genomics. 2014;103: 154–159. https://doi.org/10.1016/j.ygeno.2014.01.002

54. Xia, E.-H., Yang, D.-R., Jiang, J.-J., Zhang, Q.-J., Liu, Y., Liu, Y.-L., Zhang, Y., Zhang, H.-B., Shi, C., Tong, Y., Kim, C.-H., Chen, H., Peng, Y.-Q., Yu, Y., Zhang, W., Eichler, E.E., Gao, L.-Z. The caterpillar fungus, *Ophiocordyceps sinensis*, genome provides insights into highland adaptation of fungal pathogenicity. Sci. Rep. 2017;7: 1806. DOI:10.1038/s41598-017-01869-z
55. Grabherr, M.G., Haas, B.J., Yassour, M., Levin, J.Z., Thompson, D.A., Amit, I., Adiconis, X., Fan, L., Raychowdhury, R., Zeng, Q., Chen, Z.-H., Mauceli, E., Hacohen, N., Gnirke, A., Rhind, N., di Palma, F., Birren, B.W., Nusbaum, C., Lindblad-Toh, K., Friedman, N., Regev, A. Full-length transcriptome assembly from RNA-Seq data without a reference genome. Nat. Biotechnol. 2011;29(7): 644−130. https://doi.org/10.1038/nbt.1883
56. Chou, P.Y., Fasman, G.D.. Prediction of the secondary structure of proteins from their amino acid sequence. Adv. Enzymol. Relat. Areas. Mol. Biol. 1978;47: 45–148. DOI: 10.1002/9780470122921.ch2
57. Deleage, G., Roux, B. An algorithm for protein secondary structure prediction based on class prediction. Protein Engineering, Design and Selection 1987;1: 289–294. https://doi.org/10.1093/protein/1.4.289
58. Nakamura, N., Tanaka, C., Takeuchi-Kaneko, Y. Transmission of antibiotic-resistance markers by hyphal fusion suggests partial presence of parasexuality in the root endophytic fungus *Glutinomyces brunneus*. Mycol. Progress 2019;18: 453–462. https://doi.org/10.1007/s11557-018-1455-9
59. Zhang, Y.-J., Zhang, S., Li, Y.-L., Ma, S.-L., Wang, C.-S., Xiang, M.-C., Liu, X., An, Z.-Q., Xu, J.-P., Liu, X.-Z. Phylogeography and evolution of a fungal–insect association on the Tibetan Plateau. Mol. Ecol. 2014:23: 5337−5355. DOI: 10.1111/mec.12940
60. Turgeon, B.G., Yoder, O.C. Proposed nomenclature for mating type genes of filamentous ascomycetes. Fungal Genet. Biol. 2000;31: 1–5. DOI: 10.1006/fgbi.2000.1227
61. Holliday, J., Cleaver, M. Medicinal value of the caterpillar fungi species of the genus *Cordyceps* (Fr.) Link (Ascomycetes). A review. Int. J. Med. Mushrooms. 2008;10: 219–234. DOI: 10.1615/IntJMedMushr.v10.i3.30
62. Stone, R. Improbable partners aim to bring biotechnology to a Himalayan kingdom. Sci. 2010;327: 940–941.
63. Wei, J.-C., Wei, X.-L., Zheng, W.-F., Guo, W., Liu, R.-D. Species identification and component detection of *Ophiocordyceps sinensis* cultivated by modern industry. Mycosystema. 2016;35(4): 404–410.
64. Zhang, S.-W., Cen, K., Liu, Y., Zhou, X.-W., Wang, C.-S. Metatranscriptomics analysis of the fruiting caterpillar fungus collected from the Qinghai-Tibetan plateau. Sci. Sinica Vitae. 2018;48(5): 562−570.
65. Zhong, X., Gu, L., Wang, H.-Z., Lian, D.-H., Zheng, Y.-M., Zhou, S., Zhou, W., Gu, J., Zhang, G., Liu, X. Profile of *Ophiocordyceps sinensis* transcriptome and differentially expressed genes in three different mycelia, sclerotium and fruiting body developmental stages. Fungal Biol. 2018;122: 943–951. DOI: 10.1016/j.funbio.2018.05.011
66. Li, X., Wang, F., Liu, Q., Li, Q.-P., Qian, Z.-M., Zhang, X.-L., Li, K., Li, .W.-J., Dong, C.-H. Developmental transcriptomics of Chinese cordyceps reveals gene regulatory network and expression profiles of sexual development-related genes. BMC Genomics. 2019;20: 337. https://doi.org/10.1186/s12864-019-5708-z
67. Zhao, Y.-N., Zhang, J.-H., Meng, Q., Zhang, H., Zhou, G.-L., Li, M.-M., Wu, P.-P., Shu, R.-H., Gao, X.-X., Guo, L., Tong, Y., Cheng, L.-Q., Guo, L., Chen, C., Qin, Q. Transcriptomic analysis of the orchestrated molecular mechanisms underlying fruiting body initiation in Chinese cordyceps. Gene. 2020;763: 145061. https://doi.org/10.1016/j.gene.2020.145061
68. Yang, J.-Y., Tong, X.-X., He, C.-Y., Bai, J., Wang, F., Guo, J.-L. Comparison of endogenetic microbial community diversity between wild *Cordyceps sinensis*, artificial *C. sinensis* and habitat soil. Chin. J. Chin. Materia Medica. 2021;46(12): 3106–3115.
69. Peters, C., Elofsson, A. Why is the biological hydrophobicity scale more accurate than earlier experimental hydrophobicity scales? Proteins. 2014;82(9): 2190-2198. doi: 10.1002/prot.24582
70. Simm, S., Einloft, J., Mirus, O., Schleiff, E. 50 years of amino acid hydrophobicity scales: revisiting the capacity for peptide classification. Biol. Res. 2016;49(1): 31. doi: 10.1186/s40659-016-0092-5
71. Pfennig, K.S. Facultative Mate Choice Drives Adaptive Hybridization. Sci. 2007;318: 965–967. DOI: 10.1126/science.1146035
72. Du, X.-H., Wu, D.-M., Kang, H., Wang, H.-C., Xu, N., Li, T.-T., Chen, K.-L. Heterothallism and potential hybridization events inferred for twenty-two yellow morel species. IMA Fungus. 2020;11: 4. https://doi.org/10.1186/s43008-020-0027-1
73. Hėnault, M., Marsit, S., Charron, G., Landry, C.R. The effect of hybridization on transposable element accumulation in an undomesticated fungal species. eLife. 2020;9, e60474. DOI: https://doi.org/10.7554/eLife.60474
74. Samarasinghe, H., You, M., Jenkinson, T.S., Xu, J.-P., James, T.Y. Hybridization Facilitates Adaptive Evolution in Two Major Fungal Pathogens. Genes. 2020;11: 101. doi:10.3390/genes11010101
75. Steensels J, Gallone B, Verstrepen KJ. Interspecific hybridization as a driver of fungal evolution and Adaptation. Nat. Rev. Microbiol. 2021;19: 485–500.
76. Bennett, R.J., Johnson, A.D. Completion of a parasexual cycle in *Candida albicans* by induced chromosome loss in tetraploid strains. EMBO J. 2003;22(10): 2505–2515. DOI: 10.1093/emboj/cdg235
77. Sherwood, R.K., Bennett, R.J. Fungal meiosis and parasexual reproduction--lessons from pathogenic yeast. Current Opinion Microbiol. 2009;12(6): 599-607. DOI: 10.1016/j.mib.2009.09.005
78. Seervai, R.N.H., Jones, S.K., Hirakawa, M.P., Porman, A.M., Bennett, R.J. Parasexuality and ploidy change in *Candida tropicalis*. Eukaryotic Cell. 2013;12(12): 1629–1640. DOI: 10.1128/EC.00128-13
